# Influence of Body Mass Index (BMI) on Functional Improvements at 3 Years Following Total Knee Replacement: A Retrospective Cohort Study

**DOI:** 10.1371/journal.pone.0059079

**Published:** 2013-03-19

**Authors:** Paul Baker, Karthikeyan Muthumayandi, Craig Gerrand, Benjamin Kleim, Karen Bettinson, David Deehan

**Affiliations:** 1 Institute of Cellular Medicine, Medical School, Newcastle University, Newcastle upon Tyne, United Kingdom; 2 Department of Orthopaedic Research, Freeman Hospital, Newcastle upon Tyne, United Kingdom; 3 Freeman Hospital, Newcastle upon Tyne, United Kingdom; 4 Royal Victoria Infirmary, Newcastle upon Tyne, United Kingdom; The University of Queensland, Australia

## Abstract

**Background:**

The number of patients presenting for total knee replacement who are classified as obese is increasing. The functional benefits of performing TKR in these patients are unclear.

**Aim:**

To assess the influence pre-operative body mass index has upon knee specific function, general health status and patient satisfaction at 3 years following total knee replacement.

**Design:**

Retrospective comparative cohort study using prospectively collected data from an institutional arthroplasty register.

**Methods:**

1367 patients were assessed using the Western Ontario and McMaster University Osteoarthritis Index (WOMAC) and Medical Outcomes Trust Short Form-36 (SF-36) scores supplemented by a validated measure of satisfaction pre-operatively and subsequently at 1,2 and 3 year post-operatively. Comparisons were made by dividing the cohort into 4 groups based on body mass index (BMI) 18.5–25.0 kg/m^2^ (n = 253);>25.0–30.0 kg/m^2^ (n = 559);>30.0−35.0 kg/m^2^ (n = 373);>35.0 kg/m^2^ (n = 182).

**Results:**

Despite lower pre-operative, 1 and 3 year WOMAC and SF-36 scores patients with the highest BMIs >35.0 kg/m^2^ experienced similar improvements to patients with a ‘normal‘ BMI (18.5–25.0 kg/m^2^) at 1 year (Difference in WOMAC improvement = 0.0 (95%CI −5.2 to 5.2), p = 1.00) and this improvement was sustained at up to 3 years (Difference in 1 year to 3 year improvement = 2.2 (95%CI: −2.1 to 6.5), p = 1.00). This effect was also observed for the SF-36 mental and physical component scores. Despite equivalent functional improvements levels of satisfaction in the >35.0 kg/m^2^ group were lower than for any other BMI group (>35.0 kg/m^2^ = 84.6% satisfied versus 18.5–5.0 kg/m^2^ = 93.3% satisfied,p = 0.01) as was the proportion of patients who stated they would have the operation again (>35.0 kg/m^2^ = 69.6% versus 18.5–25.0 kg/m^2^ = 82.2%,p = 0.01).

**Conclusion:**

Obese and morbidly obese patients gain as much functional benefit from total knee replacement as patients with lesser body mass indexes. This benefit is maintained for up to 3 years following surgery. However, these patients are less satisfied with their knee replacement and almost a third would not have the operation again.

## Introduction

Obesity is a global epidemic [1], [2]. In England, greater than 26% of the population are now obese (Body Mass Index (BMI) ≥30 kg/m^2^) [1] mirroring findings from the rest of Europe [2]. Rates of obesity are increasing and it is predicted that by the end of 2012 greater than 30% of the population of England will be classified as obese [3]. Obesity has a detrimental effect on knee joint function [4]. The increased loads associated with increasing body mass are causative in the development of degenerative knee arthritis [4]–[7]. Growth in the proportion of the population with obesity, combined with an increased demand for knee arthroplasty as we service an increasingly elderly population, will inevitably lead to a rise in the number of obese patients requesting Total Knee Replacement (TKR).

The treatment of obese patients currently poses a dilemma for the operating surgeon. The timing of surgery, the role of bariatric surgery and the issue of whether surgeons should withhold knee replacement for patients above specific BMI thresholds is a matter of on-going debate [8]–[11]. Historically both obese (BMI ≥30 kg/m^2^) and morbidly obese (BMI ≥40 kg/m^2^) patients have suffered a higher incidence of complications [10], [11] and lower rates of implant survival (5-year survival rates = 74%) following TKR [12]. However this view has been challenged by contemporary reports of equivalent rates of complications [9], [13] and mid-term survival [14], [15] irrespective of the patient’s pre-operative BMI. These reports have also suggested that morbidly obese patients achieve the same functional improvements (Oxford Knee Score, Euroqol-5D) as patients with a “normal” BMI (BMI 18.5 to 25 kg/m^2^) [9]. However the validity of this conclusion is limited by a short duration of follow up (median 7 months) and it therefore remains unclear whether this finding is generalisable to longer term functional outcomes.

The financial and resource burdens associated with elective surgical procedures mean it is imperative that operations remain cost-effective irrespective of the patient cohort presenting for surgery. Central to this is the ability to demonstrate sustained improvements in the patient’s functional level and quality of life following surgery. For obese and morbidly obese patients undergoing TKR this information is currently lacking. We therefore aimed to address this by assessing the influence pre-operative BMI has upon 1) knee specific function 2) general health status and 3) patient satisfaction up to 3 years following TKR.

## Materials and Methods

### Ethics Statement

The Freeman Joint Registry is an on-going clinical audit which commenced July 2003 as a mechanism for monitoring outcomes following hip and knee replacement. Prior to surgery all patients receive a patient information sheet and informed consent is obtained covering the collection, storage and subsequent analysis of data. The project was registered with the institutional research board (Project ID number: 3290). This analysis was covered by the terms of the registry and was performed on anonomised data without need for additional patient contact. It was therefore performed as a service evaluation without need for formal ethical approval. The study was conducted in accordance with the declaration of Helsinki and the guidelines for good clinical practice.

This analysis was performed as a retrospective comparative cohort study using prospectively collected data from a single centre institutional arthroplasty register. The registry routinely collects pre-operative patient demographic details (age, gender, presences of co-morbidities, self-reported height and weight) in addition to baseline functional outcomes (Western Ontario and McMaster University Osteoarthritis Index (WOMAC) [16], Medical Outcomes Trust Short Form-36 (SF-36) [17]). Pre-operative assessment was performed within 6 weeks of surgery. Post-operatively patients were reviewed annually out to 3 years using WOMAC and SF-36 scores supplemented by measures of patient reported satisfaction [18]. Post-operatively patients were reviewed in the outpatient clinic at 1,2 and 5 years. The assessments at 1 and 2 years were therefore performed in the outpatient clinic, whereas the 3 year assessment was performed using a postal questionnaire identical to questionnaires completed in the outpatient clinic at the earlier time points.

This study was interested in assessing mid-term functional outcomes and as such only those patients who had reached the threshold for 3 year review were included. All patients undergoing primary TKR that were entered onto the registry between July 2004 and August 2008 were included irrespective of the indication for surgery (n = 1902). All patients underwent cemented TKR using either Press Fit Condylar (PFC) (Depuy, Warsaw Indiana, USA) or Triathlon (Stryker, Marwah New Jersey, USA) knee implants. From this we excluded 253 patients with missing BMI data and 282 patients with missing or invalid pre-operative or 1 year post-operative WOMAC/SF-36 data. After exclusions this left a cohort of 1367 patients with a recorded BMI and complete pre-operative and 1 year post-operative data for analysis. To ensure our exclusion criteria had not biased the study cohort we compared the demographic details of this cohort with the details of those patients who were excluded. These groups were similar for patient age (p = 0.85), gender (p = 0.43) and co-morbidity score [19] (p = 0.43) suggesting that the group included in the analysis was representative of the total population of patients presenting for TKR at our institution. The mean BMI of the study cohort (29.5 kg/m^2^) was also similar to the mean BMI of the 282 patients excluded due to lack of WOMAC/SF-36 data (29.2 kg/m^2^) (p = 0.42). Of the 1367 patients with 1 year functional outcome data, 1180 (86.3%) and 1056 (77.2%) also had outcome data at 2 and 3 years respectively.

The institutional registry uses the WOMAC score [16] to assess knee specific outcomes. The WOMAC score assesses 24 elements divided into 3 subscales (pain, stiffness, function) which are combined to produce an overall measure of knee health. General health was evaluated using the SF-36 [17], a generic health measure, which assesses both physical and mental health status. For both of these outcomes the final scores were transformed to produce a 0 to 100 point scale (100 best). Using this method the minimally clinically important difference (MCID) for these two scales are at least 15 points for the WOMAC and 10 points for the SF-36 [20].

In addition to the WOMAC and SF-36 patients were also asked to complete a short self-report questionnaire evaluating their level of satisfaction alongside questions relating to quality of life and whether they would undergo knee replacement again. The satisfaction questionnaire consists of four items focusing on satisfaction with improvement in ability to perform home or yard work, ability to perform recreational activities, the extent of pain relief and overall satisfaction with joint replacement [18]. Responses to each of the items are scored using a 4-point Likert scale with using the answers ‘Very satisfied/‘Somewhat satisfied’/‘Somewhat dissatisfied’/‘Very dissatisfied’. This outcome tool has been validated for the assessment of satisfaction in patients following TKR [18]. The quality-of-life question asked patients: “How much did the knee replacement surgery improve the quality of your life?” to which there are five possible responses ‘A Great improvement’/‘A Moderate improvement’/‘A Little improvement’/‘No improvement at all’/‘The quality of life is worse’. Patients were also asked “Knowing how well you have done following your knee replacement surgery would you have the same surgery again?” with possible responses ‘Yes’/‘No’/‘Unsure’.

Prior to analysis each patient had their BMI calculated from their self-reported height and weight [21]. To allow comparison between patients with different BMIs we intended to classify the study cohort into five groups based on the World Health Organisation criteria [22] (normal BMI = 18.5 to 25.0 kg/m^2^ (n = 253); overweight>25.0 to 30.0 kg/m^2^ (n = 559); obese class I>30.0 to 35.0 kg/m^2^ (n = 373); obese class II>35.0 to 40.0 kg/m^2^ (n = 144); obese class III>40.0 kg/m^2^ (n = 38). Power calculation based on the observed distributions of the WOMAC and SF-36, the MCID for these two scores, a power of 0.8 and a significance level of 0.05 demonstrated that for a 5 group comparison we required a minimum of 49 patients in each group. As the numbers in the obese class III group were below this we decided to combine the obese class II and III groups to ensure our analysis was sufficiently powered. Patient co-morbidity was assessed using a validated, medical record-based co-morbidity instrument [19]. This tool combines responses to 12 questions concerning current and subsequent health status to produce a co-morbidity index ranging from 0 to 24. Baseline characteristics for the four groups are given in Table 1.

**Table 1:** Patients demographics for the study cohort.

	BMI group				
Variable	Normal (BMI: 18.5 to 25.0 kg/m^2^)	Overweight (BMI: >25.0 to 30.0 kg/m^2^)	Obese class I (BMI: >30.0 to 35.0 kg/m^2^)	Obese class II & III(BMI: >35.0 kg/m^2^)	p value
n	253	559	373	182	
**Age (Years)**					
Mean (S.D)	71.1 (9.8)	70.6 (9.4)	67.2 (8.9)	64.3 (7.7)	<0.01
**Gender**					
Male: Female	116 (46%): 137 (54%)	240 (43%): 319 (57%)	157 (42%): 216 (58%)	72 (40%): 110 (60%)	0.61
**Comorbidity Index**					
Median (Range)	2 (0–11)	3 (0–16)	3 (0–12)	4 (0–12)	<0.01

Groups were compared using ANOVA for continuous variables (Age, Co-morbidity) and the Chi-Squared test for categorical variables (Gender).

### Statistical Analysis

Continuous variables were compared using one-way ANOVA with post hoc between groups comparisons performed using the Tukey honestly significant difference (HSD) test**.** Chi-squared tests were used for binary and categorical variables. The patient demographics differed between the four BMI groups (Table 1). To limit the possible confounding effects of differences in age and co-morbidity we also calculated adjusted differences in the WOMAC and SF-36 scores for these groups using the residuals of linear models including the patient demographics (age, gender and co-morbidity). SPSS version 19 (IBM corporation, Armonk, New York, USA) was used to carry out the analysis with p<0.05 considered as statistically significant.

## Results

Patient demographics for each of the four BMI categories are given in Table 1. Patients classified as obese were younger (p<0.01) and had a greater co-morbidity score (p<0.01) than patients classified as normal or overweight.

### WOMAC

For the entire study cohort the mean pre-operative and 3 year WOMAC were 36.8 (95%CI 35.9 to 37.6) and 74.4 (95%CI 73.3 to 75.4) respectively. At 1 year following surgery the mean WOMAC demonstrated a significant improvement from the pre-operative level (37.9 (95%CI 36.8 to 38.9) but thereafter the score plateaued (change between 1 and 3 years (−0.3 (95%CI −1.1 to 0.6).

**Figure 1:**
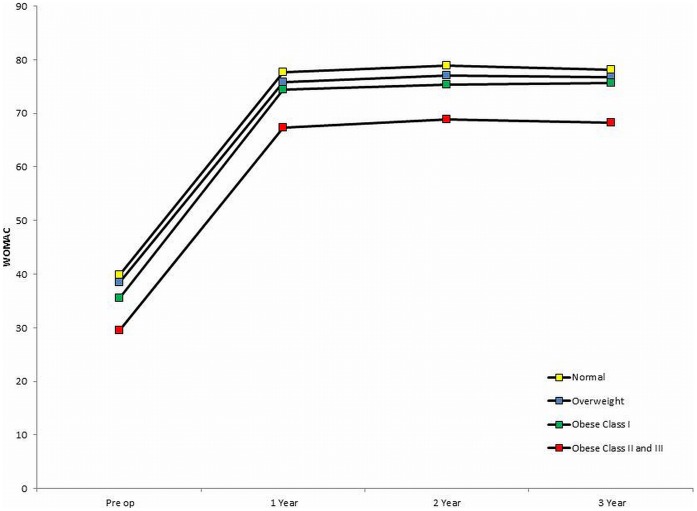
Mean WOMAC score for each of the 4 BMI groups.

A breakdown of WOMAC scores for each of the 4 BMI groups is given in table 2 and figure 1. There were significant differences in the absolute WOMAC scores between the 4 groups at each time point (all p<0.01) with a consistent trend for scores to decrease as BMI increased. Post hoc between group comparisons were made using the Tukey HSD method. This demonstrated that the mean pre-operative and 1 year post-operative WOMAC scores were significantly lower for the obese class II/III group when compared to the other three BMI groups (difference versus normal BMI: Pre-operative = 10.4 (95%CI: 6.3 to 14.4),p<0.01; 1 year = 10.3 (95%CI: 5.5 to 15.1),p<0.01; difference versus overweight: Pre-operative = 9.0 (95%CI: 5.5 to 12.6),p<0.01; 1 year = 8.4 (95%CI: 4.2 to 12.6),p<0.01; difference versus obese I: Pre-operative = 6.1 (95%CI: 2.3 to 9.8),p<0.01; 1 year = 7.1 (95%CI: 2.7 to 11.6),p<0.01). The mean WOMAC scores for the overweight group were comparable to the scores for the normal BMI group at all of the time points (p = 0.69 pre-operatively and p = 0.55 at 1 year). The obese I group had lower pre-operative scores when compared to both the normal BMI group (difference = 4.3 (95%CI: 0.9 to 7.6),p<0.01) and overweight group (difference = 2.9 (95%CI: 0.2 to 5.7),p = 0.03). However their 1 year WOMAC scores were similar (difference versus normal BMI group = 3.2 (p = 0.17), difference versus overweight group = 1.3 (p = 0.75).

**Table 2:** Comparison of the WOMAC, SF-36 PCS and SF-36 MCS scores for the 4 BMI groups.

	BMI group				
Variable	Normal (BMI: 18.5 to 25.0 kg/m^2^)	Overweight (BMI: >25.0 to 30.0 kg/m^2^)	Obese class I (BMI: >30.0to 35.0 kg/m^2^)	Obese class II & III(BMI: >35.0 kg/m^2^)	p value(ANOVA)
n	253	559	373	182	
**Mean WOMAC**					
Pre-op	39.9 (95%CI: 37.7 to 42.1)	38.5 (95%CI: 37.1 to 39.9)	35.6 (95%CI: 34.2 to 37.1)	29.5 (95%CI: 27.3 to 31.7)	<0.01
1 year	77.7 (95%CI: 75.3 to 80.1)	75.8 (95%CI: 74.2 to 77.3)	74.5 (95%CI: 72.6 to 76.4)	67.4 (95%CI: 64.4 to 70.3)	<0.01
Change Pre op to 1 year	37.8 (95%CI: 35.4 to 40.3)	37.2 (95%CI: 35.5 to 38.9)	38.9 (95%CI: 36.8 to 40.9)	37.8 (95%CI 34.8 to 40.9)	0.70
Change 1 year to 3 years	−1.7 (95%CI: −3.7 to 0.2)	−0.2 (95%CI: −1.5 to 1.2)	0.3 (95%CI: −1.3 to 1.9)	0.4 (95%CI −2.1 to 3.0)	0.40
**Mean SF-36 PCS**					
Pre-op	28.0 (95%CI: 27.0 to 28.9)	28.1 (95%CI: 27.4 to 28.7)	26.8 (95%CI: 26.1 to 27.5)	25.7 (95%CI: 24.7 to 26.8)	<0.01
1 year	37.9 (95%CI: 36.5 to 39.2)	38.0 (95%CI: 37.1 to 38.9)	36.4 (95%CI: 35.4 to 37.5)	33.0 (95%CI: 31.5 to 34.4)	<0.01
Change Pre op to 1 year	9.9 (95%CI: 8.7 to 11.1)	10.1 (95%CI: 9.1 to 10.8)	9.6 (95%CI: 8.7 to 10.6)	7.2 (95%CI: 5.8 to 8.6)	<0.01
Change 1 year to 3 years	−1.4 (95%CI: −2.6 to −0.3)	−1.2 (95%CI: −2.0 to −0.4)	−0.5 (95%CI: −1.5 to 0.4)	0.0 (95%CI: −1.6 to 1.6)	0.35
**Mean SF-36 MCS**					
Pre-op	48.1 (95%CI: 46.4 to 49.8)	48.0 (95%CI: 46.8 to 49.1)	47.1 (95%CI: 45.8 to 48.5)	42.0 (95%CI: 40.0 to 43.9)	<0.01
1 year	49.4 (95%CI: 47.9 to 51.0)	50.7 (95%CI: 49.6 to 51.7)	48.7 (95%CI: 47.3 to 50.0)	45.0 (95%CI: 43.1 to 46.9)	<0.01
Change Pre op to 1 year	1.3 (95%CI: −0.1 to 2.8)	2.7 (95%CI: 1.7 to 3.7)	1.5 (95%CI: 0.2 to 2.8)	3.0 (95%CI: 1.0 to 5.1)	0.26
Change 1 year to 3 years	−0.6 (95%CI: −2.1 to 0.9)	−2.1 (95%CI: −3.2 to −1.1)	−0.4 (95%CI: −1.6 to 0.8)	−2.1 (95%CI: −4.2 to 0.0)	0.11

p values (ANOVA) represents presence of a difference between any of the four groups.

Despite lower scores at each time point the change in the WOMAC was similar for each of the 4 groups (Table 2). Comparisons of the improvements from the pre-operative baseline to 1 year and from 1 year to 3 years demonstrated the changes in scores were marginally greater for the obese II/III groups when compared to the normal BMI group. However, this difference was not statistically significant (difference between obese II/III versus normal BMI: Pre-operative to 1 year change = 0.0 (−5.1 to 5.1),p = 1.00; 1 year to 3 year change = 2.2 (95%CI −2.0 to 6.4),p = 0.54). This finding was accentuated after adjusting the data for the differences in age, gender and co-morbidity observed between these groups using regression (Adjusted difference between obese II/III versus normal BMI: Pre-operative to 1 year change = 0.9 (−2.1 to 3.9),p = 1.00; 1 year to 3 year change = 2.6 (95%CI 0.2 to 5.0),p = 0.03). This suggests that the obese II/III patients improved to the same extent as the normal BMI patients and that the improvement was more sustained in the obese II/III group.

### SF-36

For the entire study cohort the mean pre-operative physical (PCS) and mental (MCS) component scores were 27.4 (95%CI 27.0 to 27.8) and 47.0 (95%CI 46.2 to 47.7) respectively. The corresponding 3 year scores were 36.7 (95%CI 36.0 to 37.4) and 48.5 (95%CI 47.7 to 49.3).

**Figure 2:**
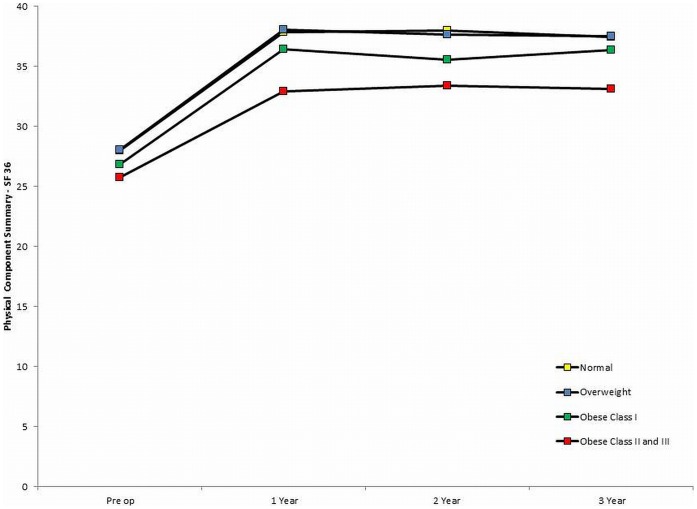
Mean SF-36 Physical Component Score for each of the 4 BMI groups.

**Figure 3:**
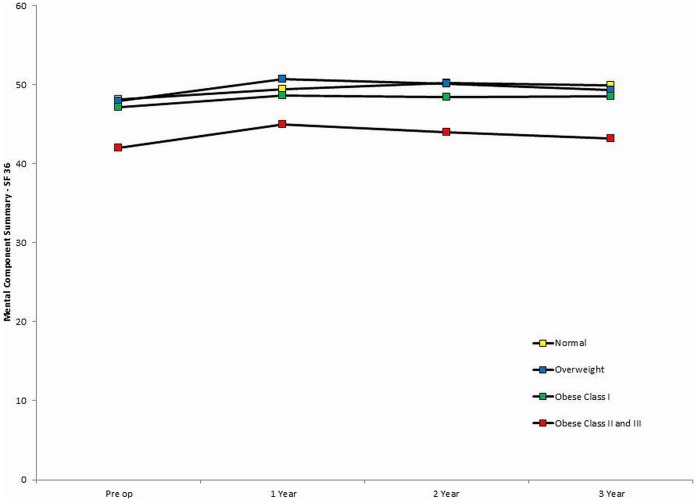
Mean SF-36 Mental Component Score for each of the 4 BMI groups.

In similarity to the WOMAC score the PCS demonstrated a significant improvement from the pre-operative level at 1 year (9.5 (95%CI 9.0 to 10.0)) with scores plateauing thereafter (change between 1 and 3 years (−0.2 (95%CI –1.0 to −0.6) (Figure 2). The observed improvements for the MCS were small and at 3 years this score had almost returned to its pre-operative level (Table 2 and Figure 3). A breakdown of PCS and MCS scores for each of the 4 BMI groups is given in table 2 which again demonstrated a significant trend for decreasing pre-operative and post-operative scores as BMI increased (both p<0.01).

In contrast to the pattern observed with the WOMAC score there was a significantly smaller improvement in the PCS at 1 year for the obese II/III group when compared to the other three BMI groups (difference versus normal BMI = 2.7 (95%CI: 0.2 to 5.2),p = 0.03; difference versus overweight = 2.8 (95%CI: 0.6 to 5.0),p<0.01; difference versus obese I = 2.4 (95%CI: 0.1 to 4.7),p = 0.04). These difference remained even after adjustment for the variation in patient demographics between the groups (adjusted difference between obese II/II versus normal BMI = 1.6 (0.8 to 2.4),p<0.01). There were no significant differences in the improvement in the PCS improvement at 1 year between the normal, overweight and obese I groups. The smaller improvements to one year were however balanced by the observation that the PCS decreased for the normal, overweight and obese I groups BMI group between 1 and 3 years but marginally improved for the obese II/III group. Therefore if the magnitude of the improvement from the pre-operative level out to 3 years was considered there were no significant differences in the PCS improvement between the 4 groups. The observed change in the MCS was small for all groups. No significant differences were observed between the groups for the MCS improvement at 1 year (p = 0.26) and between 1 and 3 years (p = 0.11) (Table 2).

### Satisfaction/Quality of Life

**Figure 4:**
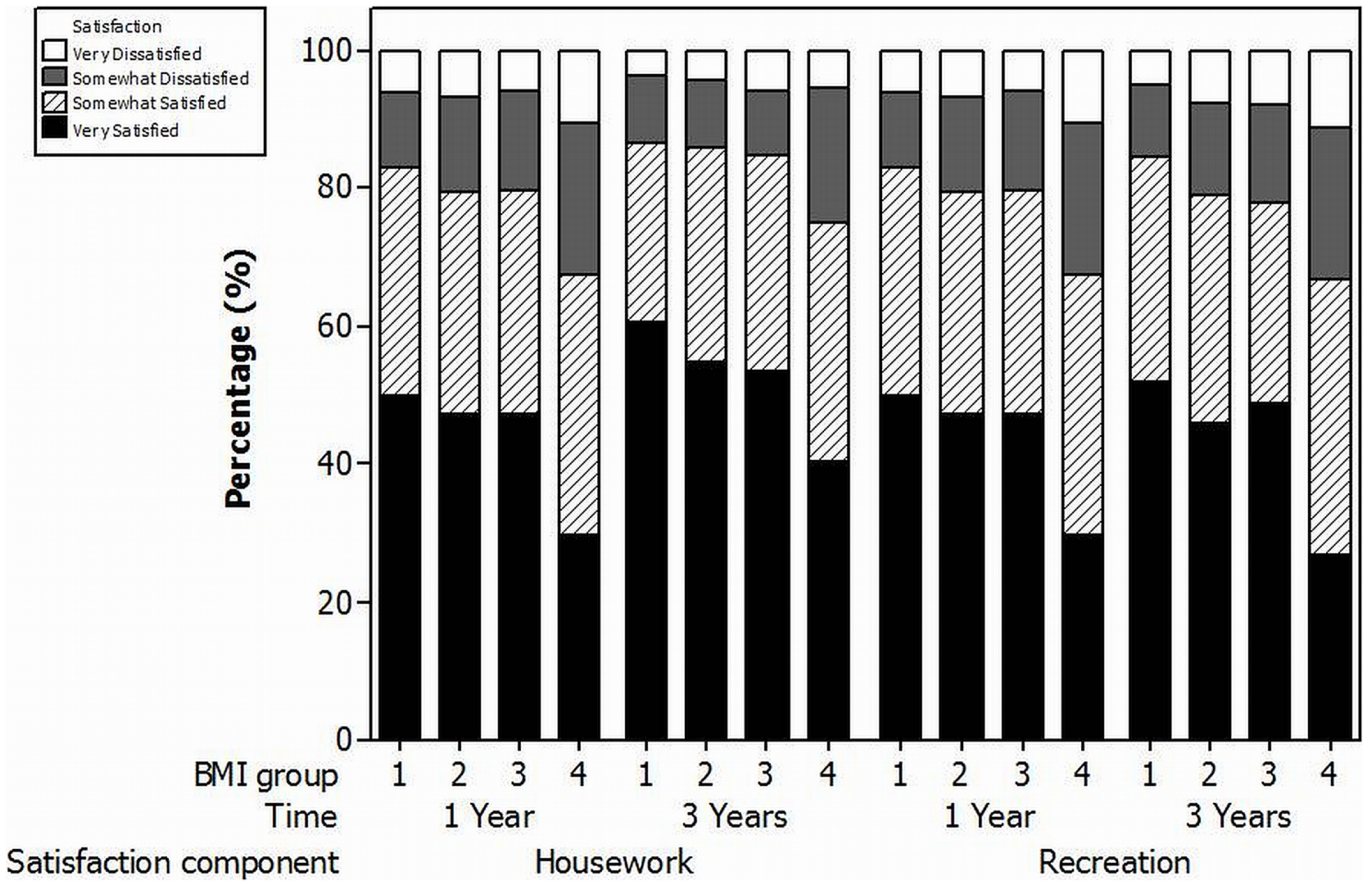
1 and 3 year satisfaction with house/yard work (Housework) and recreational activities (Recreation). Key for BMI groups: 1 = Normal BMI, 2 = Overweight, 3 = Obese class I, 4 = Obese class II/III.

**Figure 5:**
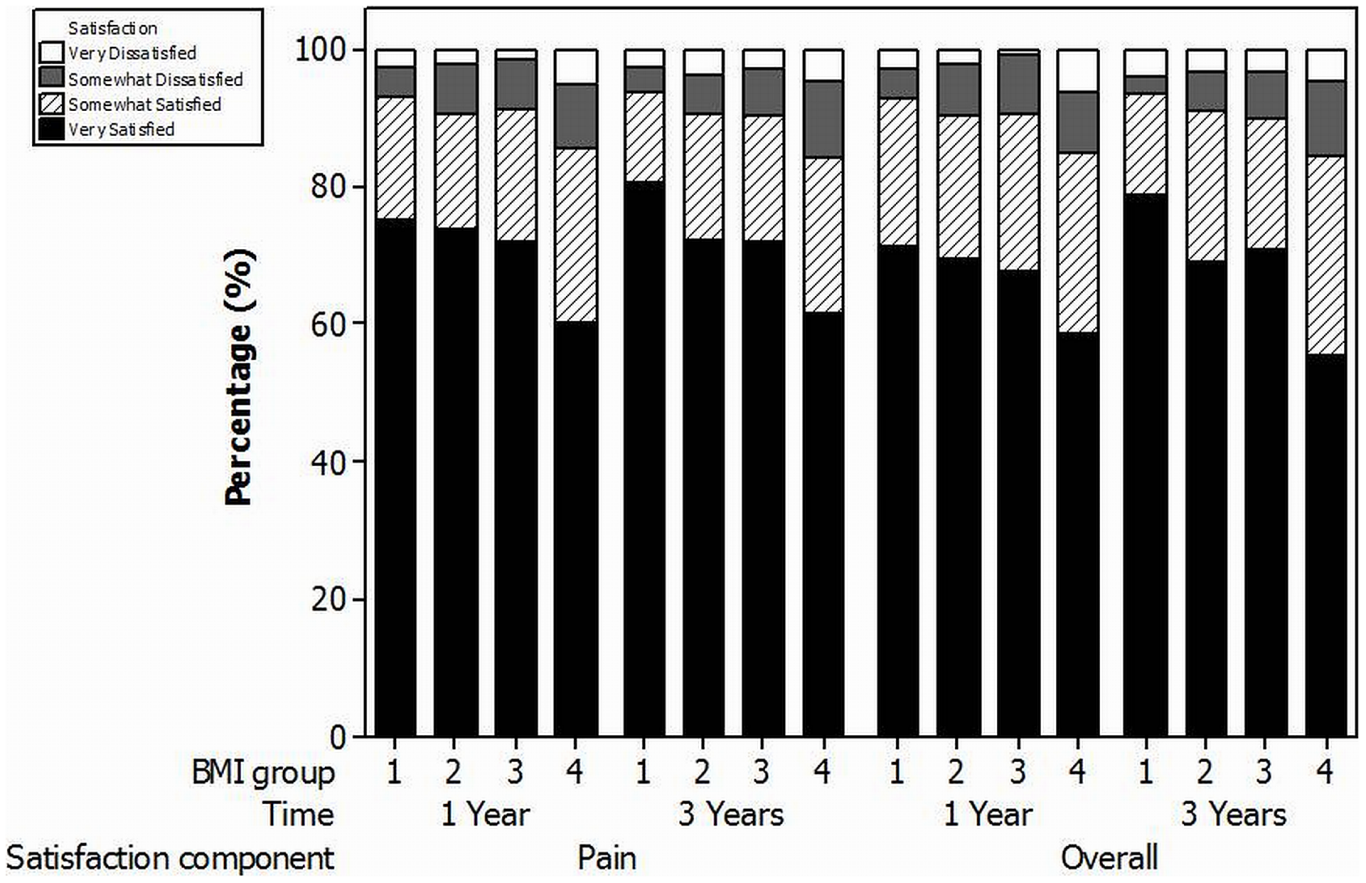
1 and 3 year satisfaction with relief of pain (Pain) and overall satisfaction (Overall). Key for BMI groups: 1 = Normal BMI, 2 = Overweight, 3 = Obese class I, 4 = Obese class II/III.

At 1 and 3 years the proportions of patients reporting they were very or somewhat satisfied with the overall result of their TKR were 90.0% and 90.4% respectively. The levels of satisfaction varied for each of the assessment modalities (house or yard work, recreational activities, pain relief, overall satisfaction) dependent upon the BMI group (Figures 4 and 5). For each modality satisfaction decreased as BMI increased with the lowest levels of satisfaction observed in the obese II/III group. At 3 years the proportion of patients reporting they were very or somewhat satisfied was significantly lower in the obese II/III groups when compared to the normal BMI group for all modalities (Satisfaction with pain relief: Normal = 93.9% versus Obese II/III = 84.0%,p<0.01; Satisfaction with house or yard work: Normal = 86.4% versus Obese II/III = 75.0%,p = 0.01; Satisfaction with recreational activity: Normal = 84.4% versus Obese II/III = 67.6%,p<0.01; Overall satisfaction: Normal = 93.3% versus Obese II/III = 84.6%,p = 0.01).

At 3 years a greater proportion of patients in the normal BMI group reported ‘great or moderate improvements’ in their quality of life when compared to the other BMI groups (Table 3). The proportion of patients reporting their quality of life was worse was highest in the obese II/III group (6.4%). Overall 77.8% of patients reported they would undergo knee replacement again but the proportion again varied dependent upon the BMI group with the highest proportion in the normal group (82.2%) and the lowest in the Obese II/III group (69.6%),p<0.01) (Table 3).

**Table 3:** Responses to questions relating to quality of life and whether patients would undergo TKR again.

	BMI group					
Assessment	Normal (BMI: 18.5 to 25.0 kg/m^2^)	Overweight (BMI: >25.0 to 30.0 kg/m^2^)	Obese class I (BMI: >30.0 to 35.0 kg/m^2^)	Obese class II & III(BMI: >35.0 kg/m^2^)	TOTAL	p value(Chi-Squared)
**Quality of Life**						
(Respondents (%))						
Great improvement	140 (71.8%)	297 (68.0%)	190 (66.2%)	63 (50.4%)	690 (66.1%)	p<0.01
Moderate improvement	34 (17.2%)	83 (19.0%)	50 (17.4%)	31 (24.8%)	198 (19.0%)	
Little improvement	8 (4.1%)	31 (7.1%)	29 (10.1%)	16 (12.8%)	84 (8.0%)	
No improvement	9 (4.6%)	17 (3.9%)	10 (3.5%)	7 (5.6%)	43 (4.1%)	
Quality of life worse	4 (2.1%)	9 (2.1%)	8 (2.8%)	8 (6.4%)	29 (2.8%)	
**Surgery again?**						
(Respondents (%))						
Yes	157 (82.2%)	336 (77.2%)	227 (79.4%)	87 (69.6%)	807 (77.8%)	p<0.01
No	13 (14.3%)	37 (8.5%)	28 (9.8%)	13 (10.4%)	91 (8.8%)	
Unsure	21 (11.0%)	62 (14.3%)	31 (10.8%)	25 (20.0%)	139 (13.4%)	

## Discussion

This study has demonstrated that patients achieve equivalent improvements in knee function and general health outcomes 3 years after TKR irrespective of their pre-operative BMI. These improvements are achieved by 1 year following surgery and are maintained at 3 years. Whilst the absolute post-operative functional scores were lower in patients classified as obese and morbidly obese the improvements they experienced were comparable to those of patients with lesser BMIs. However, despite similar functional improvements the obese and morbidly obese patients had the lowest levels of satisfaction, stated their quality of life was poorer and were less likely to undergo similar surgery again.

This study has the following limitations. Firstly BMI was calculated from patient self-reported height and weight rather than objective measures. However, previous studies have demonstrated that patient reported height and weight are accurate reflections of true height and weight in both the malnourished and overweight/obese population [23], [24]. Secondly our data did not originate from a single surgeon but instead came from an institutional registry which included data from several surgeons, introducing variability in patient selection and surgical management. Registry data was collected prospectively, however, the study was retrospective in design and some patients had to be excluded due to missing data. In addition we only collected data to 3 years and longer-term data is required to confirm the findings of this mid-term analysis. Thirdly, our institutional registry does not collect additional clinically relevant outcome data such as post-operative complications, length of hospital stay, readmission rates or radiological outcomes. Fourthly the measures of quality of life and further surgery used in this study have not been validated, which limits our ability to draw conclusions from these data. Fifthly we were not sufficiently powered to analyse the obese II and III categories separately meaning differences between patients at the top end of the BMI spectrum could not be determined, limiting the usefulness of the study in centres where surgeons restrict surgery for patients with a BMI of more than 40. Finally, we were unable to obtain data about other important factors known to influence patient satisfaction, limiting our understanding of the differences between our BMI groups. In particular, mental health scores, level of education and patient expectations are all known to influence satisfaction after TKR [25], [26]. Significant differences in the distribution of these factors between our BMI groups could therefore be a further source of confounding. Furthermore, we did not consider the impact of weight change after surgery on outcomes in this analysis.

Previous systematic reviews have reported higher mid-term failure and complication rates in obese and morbidly obese patient undergoing TKR [10], [11]. However, they have been unable to determine the effect of BMI upon functional outcomes because of a lack of published evidence [10], [11]. Recent work from the National Joint Registry for England and Wales (NJR) using patient reported Oxford Knee and Euroqol-5D Scores suggested that despite lower post-operative scores, patients with morbid obesity achieve equivalent functional improvements to patients with a ‘normal’ BMI. Unfortunately this analysis was based on a single assessment of physical functioning at a median of 7 months following surgery [9]. Similarly, we have also found that despite lower pre and post-operative knee specific and general health scores, overall improvements were similar irrespective of patient BMI. In addition this study importantly demonstrates that these findings are valid across different measurement tools, are maintained at up to 3 years post-surgery, do not change with repeated measurement and are consistent even after adjustment for differences in patients demographics between the BMI groups.

While all four BMI groups demonstrated clinically relevant improvements in the WOMAC score the improvements in the SF-36 physical and mental component scores were more modest. The minimally clinically important difference for the SF-36 components is 10 points [18]. At 3 years the mean improvement for all groups fell below this 10 point threshold for both components. This highlights that TKR primarily effects knee function and as such the value of this procedure should primarily be assessed by its effect on the joint rather than the patients overall well-being.

The obese Class II/III group reported the lowest levels of satisfaction and quality of life compared to other BMI groups. This finding was consistent across all four satisfaction domains (house or yard work, recreational activities, pain relief, overall satisfaction). This contrasts with the study by Stickles et al [27] in which there was no difference in satisfaction between obese and non-obese patients but is consistent with the findings reported by Bourne et al [25] who demonstrated that BMI independently influences patient reported satisfaction after TKR. While it is likely that BMI influences patient satisfaction we advise caution when interpreting the presented data. Factors including mental health status/depression [28], [29]; general health status [29]; need for further surgery [25], [28] and patient expectations [25] are all known to influence patient satisfaction. As we could not measure and adjust for these factors they may be a source of confounding. One explanation for the lower satisfaction observed with increasing BMI may be that satisfaction is more closely related to the absolute post-operative functional level rather than the magnitude of any improvement. The decline in the rates of satisfaction mirrored the trends for decreasing post-operative WOMAC and SF-36 scores with increasing BMI lending credence to this idea. Additionally a number of previous studies have also demonstrated a close associated between post-operative scores and patient satisfaction [25], [26].

In conclusion, this study has confirmed that obese and morbidly obese patients gain as much functional benefit from TKR as patients with lesser BMIs and that this benefit is maintained for up to 3 years following surgery. However, these patients had lower absolute post-operative functional scores, were less satisfied and had poorer quality of life ratings. In addition almost a third of this patient group would not have the operation again. Contemporary literature suggests complication and mid-term revision rates following TKR are similar for obese and morbidly obese patients. It therefore seems appropriate to pursue knee replacement for this group as long as patients are made aware that they will not achieve the same level of function and satisfaction as patients with lesser BMIs.
